# Antagonistic interaction between Nodal and insulin modulates pancreatic β-cell proliferation and survival

**DOI:** 10.1186/s12964-018-0288-0

**Published:** 2018-11-08

**Authors:** Junfeng Li, Zhihong Wang, Liwei Ren, Linling Fan, Wenjuan Liu, Yaojing Jiang, Harry K. Lau, Rui Liu, Qinghua Wang

**Affiliations:** 1 Department of Endocrinology and Metabolism, Huashan Hospital, Fudan University, Shanghai, China; 2 Department of Endocrinology, Renmin Hospital of Wuhan University, Wuhan, China; 3 Division of Endocrinology and Metabolism, Keenan Research Centre for Biomedical Science of St. Michael’s Hospital, Toronto, Ontario Canada; 4 Department of Physiology and Medicine, Faculty of Medicine, University of Toronto, Toronto, Ontario Canada

**Keywords:** Nodal, Insulin, Diabetes, β-Cell, Autocrine

## Abstract

**Background:**

Insulin signaling pathway in β-cell is essential to promote β-cells proliferation and survival, while Nodal–ALK7–Smad3 signaling involves β-cells apoptosis. We attempted to address inter-relationship between Nodal and insulin in modulating β-cell proliferation and apoptosis.

**Methods:**

Using INS-1 β-cells and isolated rat islets, we examined the effects of Nodal, insulin, or the two combined on β-cell proliferation and/or apoptosis.

**Results:**

The β-cells under high-glucose or palmitate conditions showed significant up-regulation of Nodal expression and activation of its downstream signaling pathway resulted in increased cleaved caspase-3. Insulin treatment led to significantly attenuated Nodal-induced cell apoptotic pathway. Similar results were found in directly Nodal-treated β-cell that insulin could partially block Nodal-induced up-regulation of ALK7–Smad3–caspase-3 signaling pathways with significantly attenuated β-cell apoptosis. Interestingly, we found that insulin-induced Akt activation and downstream molecules including GSK-3β, β-catenin and ERK1/2 was significantly attenuated by the co-treatment with Nodal, resulted in decreased cell proliferation. Furthermore, Nodal decreased glucose-evoked calcium influx and played a negative role during glucose-stimulated insulin secretion in the β-cells. Immunocytochemistry studies showed that Nodal treatment translocated Smad3 from cytosol mostly to the nucleus; however, co-treatment with insulin significantly decreased Smad3 nuclear localization. Co-immunoprecipitation experiments showed a directly interaction between Smad3 and Akt, and this interaction was enhanced by co-treatment with insulin.

**Conclusions:**

Our data suggest that the antagonistic interaction between Nodal and insulin has a role in the regulation of β-cell mass and secretion.

**Electronic supplementary material:**

The online version of this article (10.1186/s12964-018-0288-0) contains supplementary material, which is available to authorized users.

## Background

The homeostasis of islet β-cell mass under physiological and pathophysiologic circumstances is maintained in a balance of β-cell growth and apoptosis [1]. Excessive apoptosis leading to remarkable loss of β-cell mass is a major cause for the development of diabetic hyperglycemia [2]. β-cell apoptosis can be induced by multiple stressors such as hyperglycemia, hyperlipemia and pro-inflammatory cytokines [2]. However, the interactive role of β-cell autocrine factors on the growth, apoptosis and function of the β-cell itself is not fully understood.

Insulin exerts autocrine proliferative and anti-apoptotic effects in pancreatic β-cells [3]. The importance of autocrine insulin action is evident by observations that mice lacking insulin receptors in β-cells are glucose intolerant due to loss of compensatory capability in expanding β-cell mass in response to increased demand for insulin to maintain glucose homeostasis [4–6]. Activation of the Akt signaling pathway is critical for mediating autocrine insulin action on the β-cell to maintain appropriate mass and insulin production [7]. Defects in this signaling pathway cause impaired insulin secretion and reduced β-cell mass, supporting the perception that β-cells use a mechanism involving insulin signaling pathways for their expansion [8].

Nodal is a member of the TGF-β superfamily and induces β-cell apoptosis through the activation of the ALK7–Smad3–caspase-3 signaling pathway, while suppressing Akt signaling pathway responsible for cell growth and survival, as demonstrated in our previous reports [9, 10]. Within a rodent islet, Nodal, which is specifically expressed only in the β-cell and not in the α-cell [9] is found to be upregulated upon challenging β-cells with high glucose, palmitate, or cytotoxic cytokines, suggesting its important role in inducing and/or mediating stressor-induced apoptosis in the β-cells.

Given that insulin has anti-apoptotic effects while Nodal has pro-apoptotic effects, and that activation of Akt down-regulates the phosphorylation and nuclear translocation of Smad3 [11], we hypothesize that an antagonistic interaction between Nodal and insulin plays a role in the regulation of β-cell survival and function. In the present study, we aimed to investigate the potential mechanism by which Nodal and insulin, the two β-cell autocrine factors interplay with each other, and thus exert regulatory effects in modulating β-cell growth, apoptosis and function, using clonal INS-1 β-cells and isolated rat islets.

## Methods

### Reagents, cells and islets

Recombinant mouse Nodal protein was purchased from R&D Systems (Minneapolis, MN, USA). Recombinant human insulin was obtained from Sigma-Aldrich (St Louis, MO, USA). Palmitate (Sigma) was dissolved in serum-free RPMI 1640 (Invitrogen, Carlsbad, CA, USA) containing 1% fatty acid-free BSA (Sigma). INS-1 cells were maintained in RPMI 1640 plus 10% fetal bovine serum (FBS) as described previously [9, 10]. Rat islets were isolated from male Sprague-Dawley rat pancreas (SLAC Laboratory Animal, Shanghai, China) and cultured in RPMI 1640 medium prior to the experiments [9, 10]. The procedures of animals complied with guidelines approved by the Animal Care and Use Committee of the Shanghai Medical College, Fudan University.

### INS-1 cells and rat islets culture

For high-glucose or palmitate treated culture, both INS-1 cells and islets were cultured in serum-free RPMI 1640 medium with 30 mM glucose or 0.4 mM palmitate for 24 h, in the presence or absence of 100 nM insulin. For Nodal-treated culture, INS-1 cells were cultured in serum-free RPMI 1640 medium subjected to Nodal treatment at 1 μg/ml dosage for 24 h [9], with or without 100 nM insulin. For insulin-stimulated phosphorylation assay, insulin (100 nM) treatment was applied at the end of the experiment for 10 min (Fig. 5) [10].

### Western blot analysis

Both INS-1 cells and rat islets were lysed in RIPA lysis buffer, 25 μg of protein was loaded and resolved by SDS-PAGE followed by semidry transfer to nitrocellulose membranes [9]. The primary antibodies used were as follows: Smad3 (1:1000), phosphorylated Smad3 (p-Smad3) (1:1000), Akt (1:1,000), p-Akt (1:1,000), ERK1/2 (1:1,000), p-ERK1/2 (1:1,000), β-catenin (1:1,000), p-β-catenin (Ser-675) (1:1,000), caspase-3 (1:1,000) and cleaved caspase-3 (1:1,000) were obtained from Cell Signaling (Danvers, MA, USA); Nodal (1:500) and ALK7 (1:1,000) were from R&D Systems; and GSK-3β (1:1,000), p-GSK-3β (1:1,000) as well as GAPDH (1:10,000) were from Abcam (Cambridge, MA, USA). Protein band densities were quantified using the ImageJ program, and data were normalized to control [9].

### Cell proliferation assay

Cell proliferation was measured using a Cell Proliferation BrdU-Elisa kit (Cat. No. 11647229001, Roche, Mannheim, Germany). INS-1 cells cultured in RPMI 1640 medium containing 10% FBS at a density of 2 × 10^3^ cells/well on 96-well plates were treated with medium alone, or with 1 μg/ml Nodal in the presence or absence of 100 nM insulin up to 72 h. Before harvesting, INS-1 cells were incubated with 5-bromo-2′-deoxyuridine (BrdU) for 2 h. The pyrimidine analogue incorporation into DNA was measured using the colorimetric cell proliferation ELISA according to the manufacturer’s instructions.

### Cell apoptosis assay

Cell apoptosis was measured by flow cytometry analysis. INS-1 cells were cultured in serum-free RPMI 1640 medium alone or with 1 μg/ml Nodal, in the presence or absence of 100 nM insulin for 24 h. Cells were then harvested at a density of 2 × 10^5^ cells/ml in the binding buffer and stained with the Annexin V/propidium iodide (PI) staining assay (BD Biosciences, San Jose, CA, USA) for 10 min at room temperature and analyzed using a FACSCalibur flow cytometer and CellQuest software (BD Biosciences). Apoptotic cells were identified based on Annexin V^+^ staining and can be subdivided into early apoptotic cells (PI^−^/Annexin V^+^) or late apoptotic cells (PI^+^/Annexin V^+^) [12].

### Glucose-induced insulin secretion

INS-1 cells grown in 24-well plates to 80% confluence were rinsed twice and incubated with Krebs–Ringer bicarbonate (KRB) buffer containing 115 mM NaCl, 5 mM KCl, 24 mM NaHCO_3_, 2.5 mM CaCl_2_, 1 mM MgCl_2_, 10 mM HEPES, 2.8 mM glucose and 0.1% BSA for 60 min. Then cells were treated with 16.7 mM glucose in KRB buffer at 37 °C, in the presence of various concentrations of Nodal (0, 1, 10 μg/ml) for different periods of time (0, 5, 15, 30 min). Supernatants were collected and insulin levels were detected by an insulin Elisa kit (Abcam). The insulin secretion was normalized to the cellular protein content [10].

### Cytoplasmic free Ca^2+^

To assess cytoplasmic free Ca^2+^ concentration, INS-1 cells were loaded with 4 μM fura-2/acetoxymethylester (Molecular Probes) at 37 °C for 30 min. Changes in [Ca^2+^]_I_ were measured with a RF-5000 fluorescence spectrometer (Shimzau, Kyoto, Japan). The emissions of two excitation wave lengths of 340 and 380 nm were used to calculate fluorescent ratio (F340/F380), representing changes in [Ca^2+^]_I_.

### Immunocytochemistry

INS-1 cells treated with medium alone, or with 1 μg/ml Nodal in the presence or absence of 100 nM insulin for 15 min (Additional file 1: Figure S1) or for 24 h (Fig. 7) were fixed, permeablized, and blocked [9], and incubated with relevant antibodies: Akt and p-Akt (1:400, Cell Signaling); or Smad3 and p-Smad3 (1:500, Abcam) at 4 °C overnight. The corresponding Alexa- or Cy3-conjugated secondary antibodies were used. Images were obtained using a Nikon inverted fluorescence microscope.

### Co-immunoprecipitation

Co-IP studies were performed in cell lysate of INS-1 cells (100 μg) treated with or without insulin (100 nM, 15 min) using anti-Smad3 (Abcam 75512) or anti-p-Akt (Cell Signaling 12694S) antibodies overnight at 4 °C followed by immunoprecipitation using protein A/G-sepharose bead slurry. The antigen-antibody complex was eluted from the beads and analyzed by Western-blot.

### Statistical analysis

All data were presented as mean ± SE of independent experiments, and analyzed by using unpaired Student *t* test or One-way ANOVA with Tukey post-hoc test as appropriate. Significance was assumed at a *P* value < 0.05.

## Results

### Insulin decreases high-glucose- or palmitate-induced apoptosis through reducing nodal–ALK7–p-Smad3 expression

**Fig. 1:**
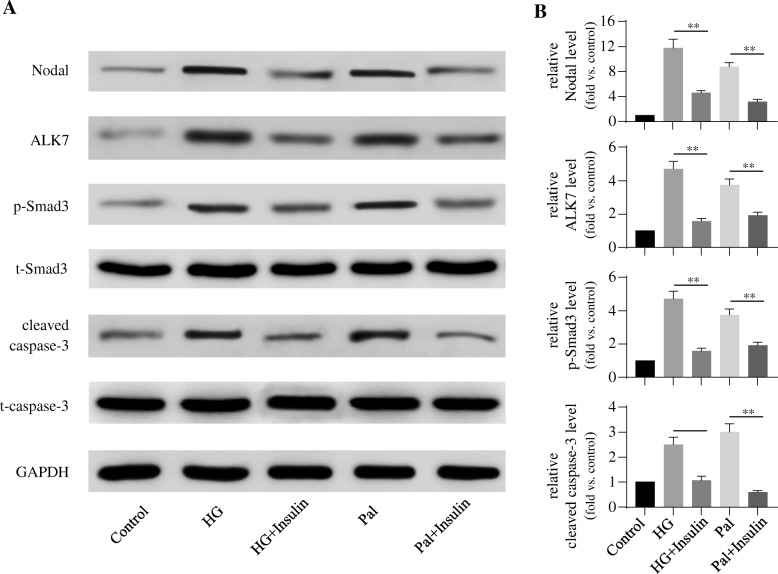
Insulin protected high-glucose- or palmitate-induced INS-1 cell apoptosis via down-regulation of Nodal–ALK7–p-Smad3 expression. INS-1 cells were cultured in serum free medium and treated with medium alone (Control), or with 30 mM glucose (HG) or 0.4 mM palmitate (Pal) for 24 h (with or without 100 nM insulin) **a**: Cell lysates were subjected to western blot analysis using relevant antibodies as indicated. **b**: Bar graphs represent densitometry analysis, data were normalized to control and expressed as mean ± SE. *n* = 3. **, *p* < 0.01. t-Smad3: total Smad3; t-caspase-3: total caspase-3

**Fig. 2:**
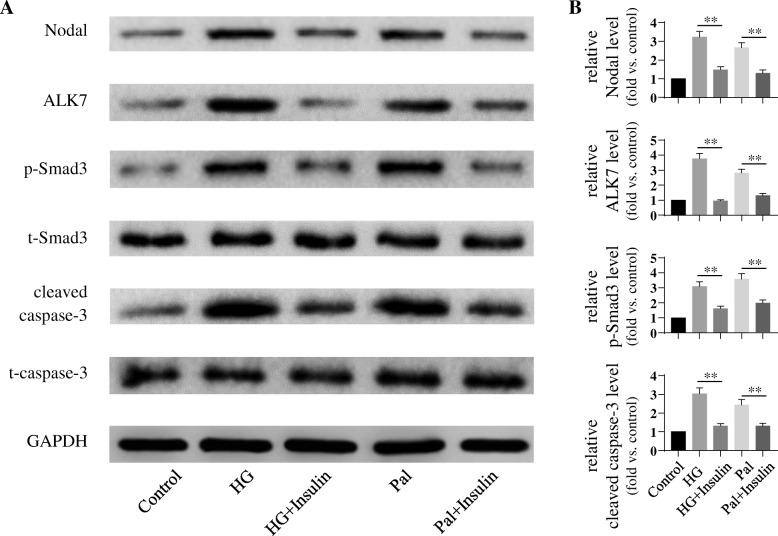
Insulin attenuated high-glucose- or palmitate-induced apoptosis and Nodal–ALK7–p-Smad3 expression in Sprague-Dawley rat islet cells. Isolated rat islet cells were treated in serum free medium alone (Control), or with 30 mM glucose (HG) or 0.4 mM palmitate (Pal) for 24 h in the presence or absence of 100 nM insulin. **a**: Cell lysates were subjected to western blot analysis using relevant antibodies as indicated. **b**: Bar graphs represent densitometry analysis, data were normalized to control and expressed as mean ± SE. *n* = 3. **, *p* < 0.01

To examine whether insulin protected stress-stimulated β-cell apoptosis and if this is through the modulation of Nodal–ALK–Smad3 pathway, we conducted western blotting analysis in the INS-1 cells undergoing apoptosis induced by high glucose or palmitate in the presence or absence of insulin. Cell treated with high-glucose and palmitate showed significant elevated Nodal–ALK7–p-Smad3 expression led to increased cleaved caspase-3 protein level when compared to control group (Fig. 1). However, these cell apoptotic effects were significantly attenuated by insulin treatment (Fig. 1). These observations were further determined in primary islet cell culture. Isolated rat islets under the high-glucose or palmitate treatment showed high protein level of cleaved caspase-3, which was associated with elevated Nodal, ALK7, and p-Smad3 protein expression (Fig. 2). Given insulin treatment on these islets showed significantly down-regulation of Nodal–ALK7–p-Smad3 signaling pathway with the reduction of cleaved caspase-3 expression when compared to no insulin-treated groups, and nearly reached control group (Fig. 2). These results suggest that high-glucose or palmitate induces β-cell apoptosis through enhancing Nodal–ALK7–p-Smad3 signaling pathway, and insulin exerts anti-apoptotic effects through attenuating Nodal and its down-stream signaling pathway.

### Insulin inhibits nodal-induced cell apoptosis via down-regulation of ALK7–p-Smad3 pathway

**Fig. 3:**
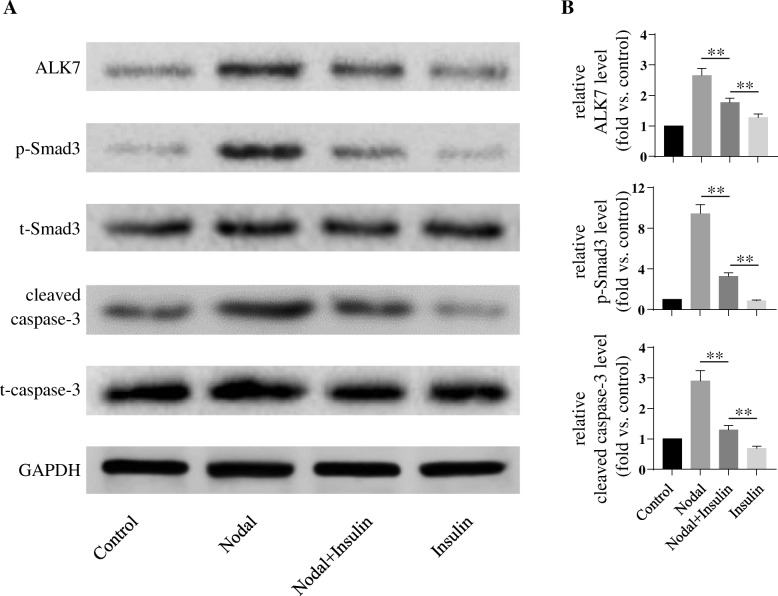
Insulin decreased Nodal-induced INS-1 cells apoptosis. INS-1 cells were cultured in serum free medium and treated with medium alone (Control) or with 1 μg/ml Nodal for 24 h (with or without 100 nM insulin). **a**: Cell lysates were subjected to western blot analysis using relevant antibodies as indicated. **b**: Bar graphs represent densitometry analysis, data were normalized to control and expressed as mean ± SE. *n* = 3. **, *p* < 0.01

**Fig. 4:**
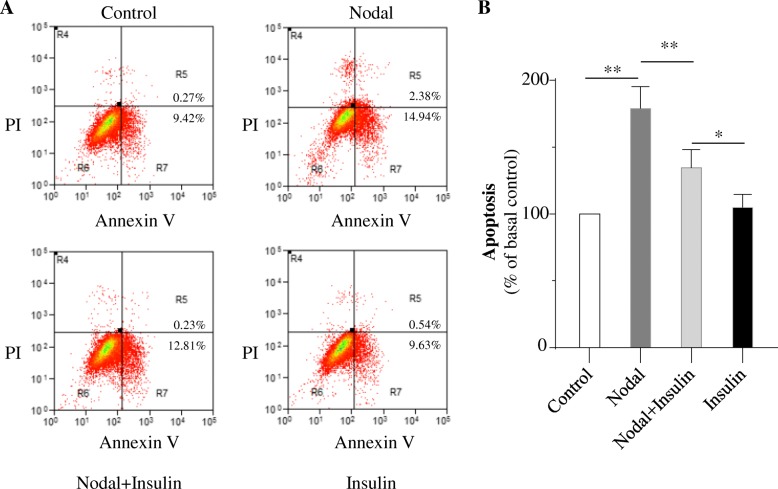
Nodal induced INS-1 cells apoptosis, but insulin suppressed apoptosis. **a**: INS-1 cells were staved in serum free medium and treated with medium alone (Control) or with 1 μg/ml Nodal (with or without 100 nM insulin) for 24 h. Cell apoptosis was assessed by flow cytometry. Apoptotic cells were identified based on Annexin V^+^ staining and could be subdivided into early apoptotic cells (PI^−^/Annexin V^+^) or late apoptotic cells (PI^+^/Annexin V^+^). **b**: Bar graphs represent apoptosis (Annexin V^+^) in A. The apoptosis was presented as percent of control values. In the histogram, the first column represents the basal control (=100%). *n* = 3. **, *p* < 0.01

To further examine whether insulin could attenuate Nodal-induced β-cell apoptosis, INS-1 β-cells were directly treated by Nodal for 24 h with or without insulin (Fig. 3). We found that Nodal-treated cells showed highly activation of ALK7–p-Smad3 pathway with significantly increased cleaved caspase-3 protein levels when compared to control groups. This Nodal-induced cell apoptotic pathway was significantly reduced in the INS-1 cells co-cultured with 100 nM insulin (Fig. 3). Furthermore, flow cytometry cell apoptosis assay determined that Nodal-induced apoptosis was largely reduced in INS-1 cells when co-treated with insulin (Fig. 4). It was noted that, although the treatment of insulin did not affect cell apoptosis under basal condition, the rate of Nodal-induced apoptosis was significantly decreased in the presence of insulin (Figs. 3 and 4), suggesting that Nodal-induced cell apoptotic pathway could be partially blocked by insulin. Taken together, these findings suggested that Nodal-induced β-cell apoptosis through the activation of ALK7–p-Smad3 signaling pathway could be inhibited, at least in part, by insulin, indicating an antagonistic interaction between Nodal and insulin in the regulation of INS-1 cells survival.

### Insulin-induced phosphorylation of Akt, GSK-3β, ERK1/2 and β-catenin is diminished by nodal

**Fig. 5:**
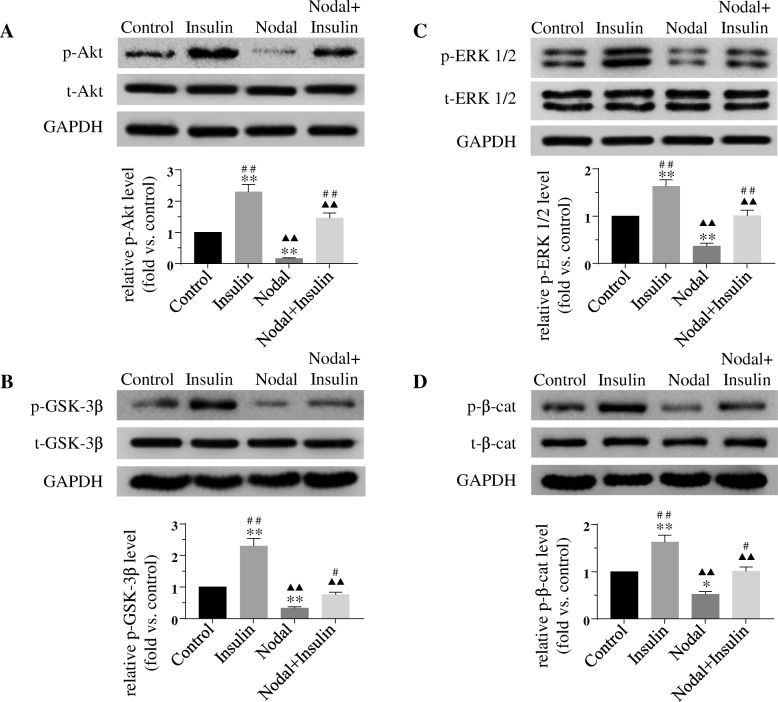
Insulin stimulated-phosphorylation of Akt, GSK-3β, ERK1/2 and β-catenin (Ser-675) is diminished by Nodal. INS-1 cells were cultured in serum free medium with or without Nodal (1 μg/ml) treated for 24 h, and then cells were exposed to insulin (100 nM) or not for 10 min. Western blotting detection of (**a**) phosphorylated and total Akt, (**b**) phosphorylated and total GSK-3β, (**c**) phosphorylated and total ERK1/2, (**d**) phosphorylated and total β-catenin, and relative protein expression levels. Bar graphs represent densitometry analysis, data were normalized to control and expressed as mean ± SE. *n* = 3. *, *p* < 0.05; **, *p* < 0.01, vs. Control. ^▲▲^, *p* < 0.01, vs. Insulin. ^#^, *p* < 0.05; ^##^, *p* < 0.01, vs. Nodal

To determine whether insulin-induced phosphorylation of Akt, GSK-3β, ERK1/2 and β-catenin (Ser-675) were affected by Nodal treatment, INS-1 cells were pretreated with or without 1 μg/ml Nodal for 24 h, and then treated with or without 100 nM insulin for 10 min. Western blotting analysis detected that insulin treatment increased the phosphorylation of Akt, GSK-3β, ERK1/2 and β-catenin, whereas this insulin effect was partially reduced in the cells pre-treated with Nodal (Fig. 5). These results suggest that Nodal exerts antagonistic effects on insulin-induced activation of signaling pathways involving Akt, GSK-3β, ERK and β-catenin in the β-cells.

**Fig. 6:**
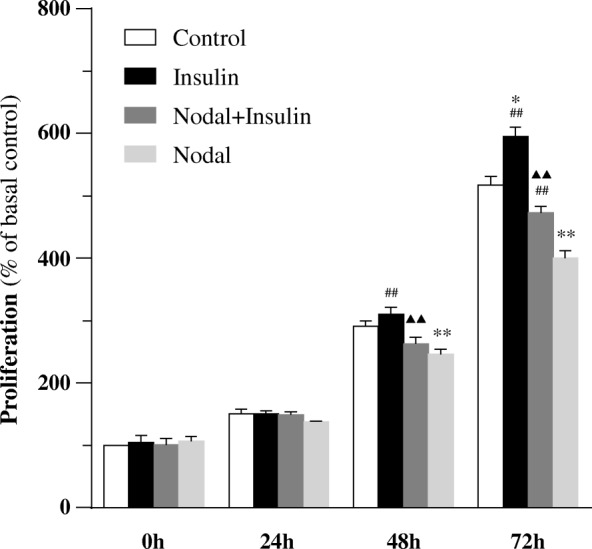
Insulin increased proliferation of INS-1 cells, but Nodal decreased proliferation. INS-1 cells were treated with culture medium alone or with 100 nM insulin,in the presence or absence of 1 μg/ml Nodal for 0–72 h. Cell proliferation was assessed by Brdu-Elisa. The proliferation was presented as percent of basal control absorbance values. In the histogram, the first column represents the basal control (=100%). *n* = 4. *, *p* < 0.05; **, *p* < 0.01, vs. control. ^▲^, *p* < 0.05; ^▲▲^, *p* < 0.01, vs. insulin. ^#^, *p* < 0.05; ^##^, *p* < 0.01, vs. Nodal

To determine whether Nodal exerts anti-proliferation effects and insulin exerts pro-proliferation effects on β-cells, we conducted proliferation assay using Brdu-Elisa kit in INS-1 cells. It was noted that Nodal-treated cells showed significantly decreased cell proliferation at either 48 h or 72 h when compared to control (Fig. 6). However, co-treatment of insulin significantly attenuated this Nodal effect was occurred at 72 h (Fig. 6). It was noted that under basal condition insulin alone significantly increased β-cell proliferation, which was attenuated in the cells co-treated with Nodal. These observations suggest that insulin could partially rescue Nodal-induced decreases in cell proliferation, potentially through activating signaling pathway involving Akt/GSK3β and ERK activation (Fig. 5).

### Co-treatment with insulin decreased nodal-induced Smad3 nuclear translocation

**Fig. 7:**
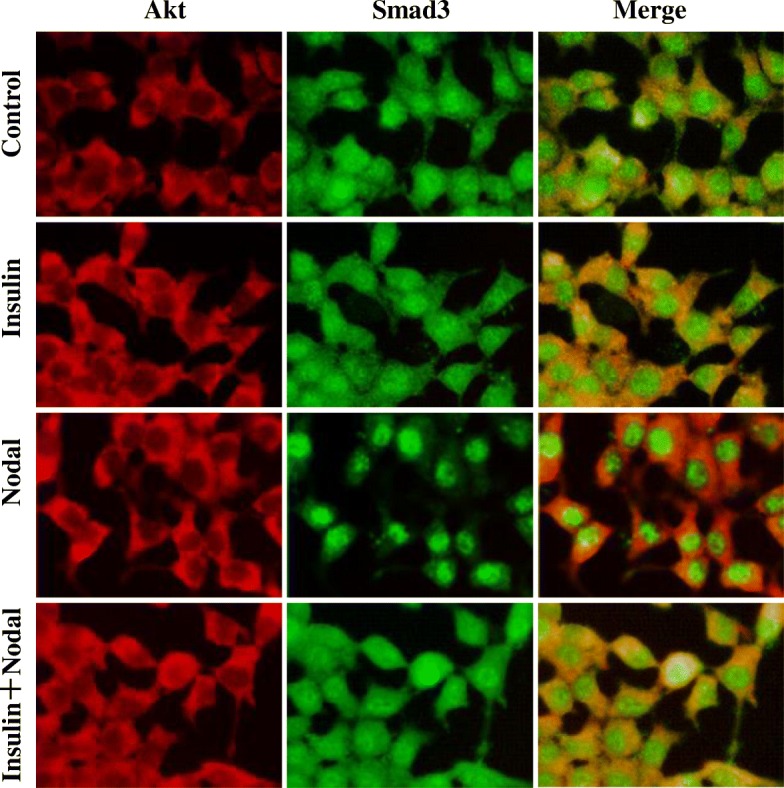
Insulin attenuated Nodal-induced nuclear translocation of Smad3. INS-1 cells were treated with culture medium alone or with 100 nM insulin,in the presence or absence of 1 μg/ml Nodal for 24 h. Cells were fixed and stained with anti-Akt or anti-Smad3, and examined by fluorescence microscopy

As a superfamily member of TGF-β, Nodal has been previously showed to induce β-cell apoptosis through activation of signaling pathway involving Smad3 phosphorylation [9]. Immunocytochemistry (ICC) studies showed that Smad3 proteins are localized in both cytosolic and nuclear compartments, and phosphorylated Smad3 are mostly localized in the nucleus (Additional file 1: Figure S1). While 15 min Nodal treatment did not change its localization pattern, 24 h Nodal treatment translocated Smad3 from cytosol compartments mostly to the nuclear localization (Fig. 7). However, co-treatment with insulin significantly decreased Smad3 nuclear localization (Fig. 7). This suggests that interaction of Akt and Smad3 occurs under basal conditions, treatment of insulin (24 h) prevented activation of Smad3 proteins in the nucleus.

### Smad3 and Akt were physically interacted

**Fig. 8:**
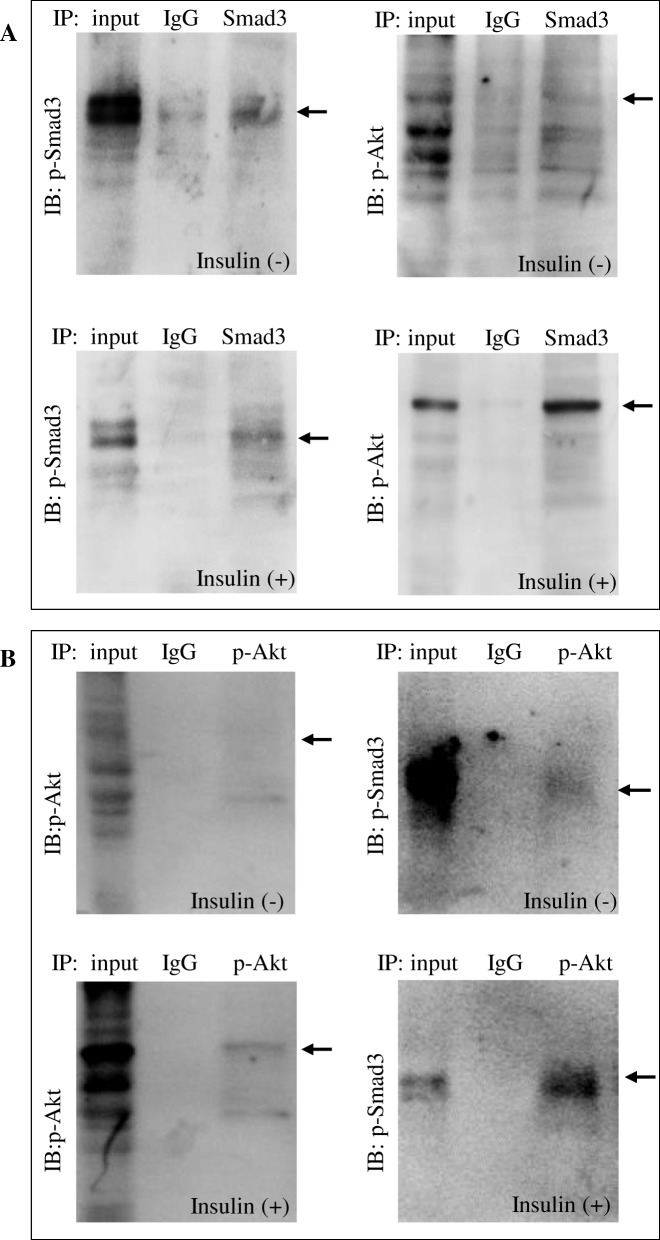
Akt directly interacted with Smad3. **a**: The left two shows p-Smad3 protein from cell lysates with or without insulin (100 nM, 15 min) was pulled down using Smad3 specific antibody. The right shows its reciprocal protein p-Akt was detected by p-Akt specific antibody. **b**. The left two shows p-Akt protein from cell lysates with or without insulin (100 nM, 15 min) was pulled down by p-Akt specific antibody. The right shows the p-Smad3 was detected by p-Smad3 specific antibody. p-Smad3: 56 kDa, p-Akt: 60 kDa

 To further verify whether Smad3 and Akt are physically interacted, we performed Co-IP experiments and followed by Western blot analysis in cell lysates derived from INS-1 cells using relevant antibodies. The results showed that under basal conditions, there was an interaction between Smad3 and Akt (Fig. 8), the interaction was enhanced in the INS-1 cells treated with insulin.

### Nodal attenuated glucose-induced insulin secretion and Ca^2+^ influx

**Fig. 9:**
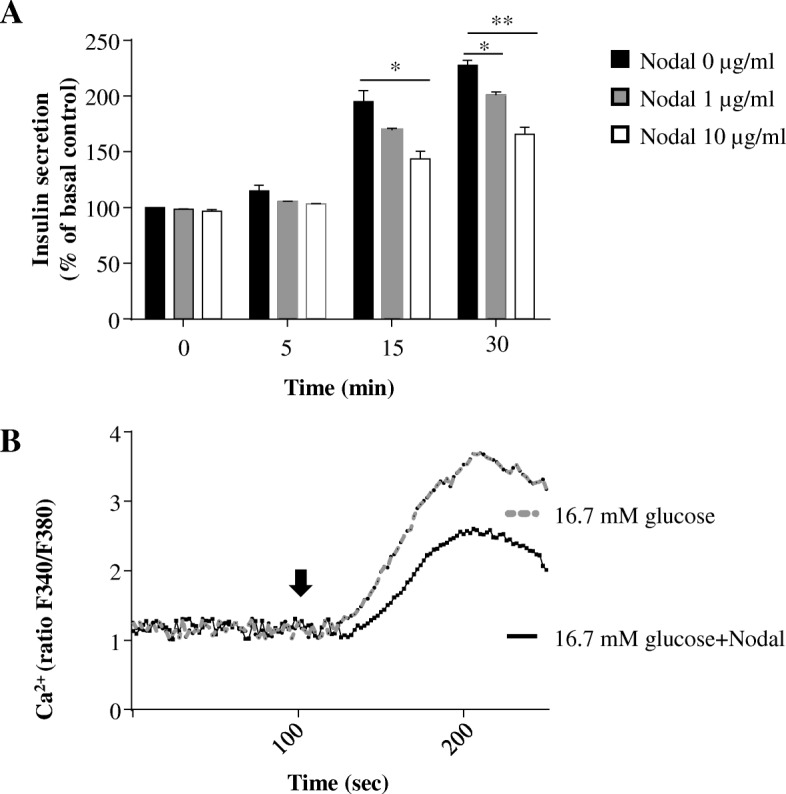
Nodal treatment decreased insulin secretion and Ca^2+^ influx stimulated by glucose in INS-1 cells. **a**: INS-1 cells were switched from 2.8 mM glucose to 16.7 mM glucose in the presence or absence of Nodal at different doses as indicated for different periods of time (0, 5, 15, 30 min). Elisa was performed to detect insulin secretion. The insulin secretion was normalized to the cellular protein content and presented as percent of basal control insulin secretion. In the histogram, the first column represents the basal control (=100%). **b**: Fura-2 loaded INS-1 cells were switched (arrow indicating the time) from 2.8 mM glucose to 16.7 mM glucose with or without 1 μg/ml Nodal. The fluorescent ratio (F340/F380) was calculated to reflect changes in Ca^2+^ influx. *n* = 3. *, *p* < 0.05; **, *p* < 0.01

 To investigate the effect of Nodal on insulin secretion, we performed glucose stimulated insulin secretion in INS-1 cells. The results showed that, when the INS-1 cells were switched from low glucose (2.8 mM) to high glucose (16.7 mM), increased insulin secretion was detected in a time dependent fashion. However, glucose-stimulated insulin secretion was markedly decreased in the cells co-treated with Nodal (Fig. 9a). Using fluorescent fura-2 Ca^2+^ indicator we measured intracellular Ca^2+^ concentrations in the INS-1 cells during the insulin secretion assay. We found that high glucose significantly potentiated Ca^2+^ influx in INS-1 cells, which was largely decreased in the cells treated with Nodal (1 μg/ml) (Fig. 9b), suggesting that Nodal and its downstream ALK7–Smad3 signaling pathway attenuated glucose-stimulated insulin secretion via impairing glucose-stimulated Ca^2+^ influx in the β-cells.

## Discussion

This study examined the β-cell autocrine factors of Nodal and insulin in regulating cell proliferation and apoptosis, and insulin secretion. Using INS-1 cells and rat islets culture, we show that, as the same as Nodal protein, high glucose or palmitate-induced upregulation of ALK7-Smad3 signaling pathway can be attenuated by insulin; whereas insulin-stimulated β-cell growth signaling pathway Akt, ERK, GSK-3β and β-catenin activation is decreased by Nodal co-treatment. The antagonistic regulation between Nodal and insulin is coupled to the cell survival and proliferation. Notably, while insulin decreases high glucose or palmitate-induced upregulation of Nodal, insulin can also diminish Nodal-induced Smad3 phosphorylation and nuclear translocation. Co-IP studies showed a direct interaction between Nodal and Akt. Furthermore, Nodal can decrease glucose-evoked calcium influx and insulin secretion in the β-cells. This study provides the first in vitro experiments to delineate the antagonistic regulation between Nodal and insulin and suggests that the imbalance of the two signaling pathways may contribute to β-cell dysfunction under pathophysiological conditions.

Autocrine insulin signaling in β-cells plays a critical role in the maintenance of appropriate β-cell mass and insulin synthesis and secretion, defect insulin signaling is associated with declined β-cell mass and the onset of diabetes, which was well-documented in vitro and in vivo studies [1, 7, 13, 14]. In rodent islets, Nodal is found to be expressed in the endocrine β-cells only, implicating its potentiality of β-cell autocrine regulation [9]. In our previous and current in vitro studies, high glucose, free fatty acids, or proinflammatory cytokines induced β-cell apoptosis is associated with activation of Nodal–ALK7–Smad3–caspase-3 and suppression of Akt signaling pathway, and co-treatment with Nodal or upregulation of Nodal receptor ALK7 through adenovirus transfection in INS-1 cells can lead to apoptosis and inhibit insulin-induced activation of the Akt signaling pathway [9, 10]. These findings suggest that Nodal signaling represent a negative regulatory machinery in the regulation of β-cell mass. This notion is consistent with previous observations that mice lacking Nodal receptor ALK7 displayed increased β-cell proliferation, enlarged β-cell mass enlargement and enhanced glucose-stimulated insulin secretion [15].

Insulin attenuated β-cell death induced by Nodal protein or high-glucose/ palmitate via down-regulation of ALK7–Smad3 and caspase-3 signaling, suggesting that activation of insulin signaling pathway could diminish Nodal-induced apoptosis pathway. Akt phosphorylation plays a key role in insulin signaling pathway in mediating insulin- and/or β-cell growth factor(s)-induced proliferation and survival [1, 16, 17]. Mice lacking active Akt in the β-cells showed inability of β-cell mass compensation and increased susceptibility to experimental diabetes [8]. Conversely, enhanced Akt activity is found to be associated with increased β-cell proliferation and β-cell mass in the Zucker fatty rats [18], which spontaneously develop obese and insulin resistance but not diabetes [19]. It is presumably that the Akt-mediated β-cell mass compensatory response represents an important mechanism allowing these obese rats remained in euglycemia despite insulin resistance [1].

Previous in vivo animal studies also suggested the role for Smad3 in mediating high-fat diet induced obesity and insulin resistance [20]. In an islet, Nodal induced Smad3 activation represents a major mechanism underlying Nodal induced-β-cell apoptosis. Our data show that 24 h Nodal treatment resulted Smad3 nuclear translocation and activation, which, however, is largely prevented by insulin co-treatment. Our observations that in the β-cells, Smad3 and Akt are physically interacted with each other, consistent with previous studies [11, 21] providing additional evidence that Akt could directly interact with Smad3 to inhibit its activation and nuclear translocation that leads to inhibition of Smad3-mediated apoptosis. The phosphorylation of Smad3 is closely related to apoptosis induced by activation of Nodal-ALK7 pathway [9, 22]. Our present studies suggests, there is a direct antagonistic relationship between the apoptosis signaling pathway represented by Nodal-ALK7-pSmad3, and the growth signaling pathway represented by insulin-Akt. In line with these findings, previous studies showed that Nodal signaling directly interacted with the insulin gene in islet β-cells, suggesting the antagonistic effects may occur at the transcription level [23].

Of note, treatment of insulin also decreases the expression of Nodal protein, implying that the antagonism may also occur at the transcription level. While the precise molecular mechanism yet to be delineated the finding that insulin downregulates Nodal expression may represent one of the potential mechanisms by which insulin exerts antagonistic effects to the Nodal signaling pathway in modulating cell proliferation and survival in the β-cells. It is conceivable that activation of insulin signaling involving Akt phosphorylation to prevent Smad3 nuclear translocation/activation, leading to decreased transcriptional activities initiated by Smad3 activation. This notion is in part supported by previous evidence that Smad3 activation could enhance the expression of multiple types of transcription factors [24, 25], and phosphorylated Smad3 mediated by ALK7 signaling pathway could also function as an activator of Nodal genes [26].

Extracellular signal regulated kinase (ERK) is a member of the mitogen activated protein kinase family, with two subtypes denoted as ERK1 and ERK2. Nuclear translocation of phosphorylated ERK can control the activation of multiple transcription factors, leading to regulation of cell proliferation and differentiation [27]. Our results show that insulin can induce phosphorylation of ERK1/2, which is consistent with previous studies [27–29], and may partially explain why insulin can promote β-cell growth [29]. We further demonstrate that Nodal-ALK7 can inhibit insulin-induced phosphorylation levels of ERK. Calcium ion influx has also been reported as a mechanism for the activation of ERK1/2 signaling pathway in islet β-cells [27]. In our study, Nodal-ALK7 activation inhibited the calcium ion influx, which may partially explain its inhibitory effect on ERK signaling. Additionally, studies have shown that phosphorylated ERK1/2 can promote insulin gene transcription in β-cells stimulated by glucose [27]. This suggests that Nodal-ALK7 activation may not only inhibit insulin secretion but also affect insulin synthesis. Further studies are warranted to demonstrate this effect.

The β-catenin/TCF7L2 dependent canonical Wnt pathway is regarded to be associated with pancreatic development, islet function and insulin synthesis/secretion [30–32]. That fact that β-catenin translocates from cytoplasm to the nucleus and acts on transcription factor TCF7L2 to regulate downstream target gene expression plays a pivotal role in the pathway [32]. When GSK-3β is phosphorylated, degradation of β-catenin by GSK-3β is inhibited, and the Wnt signaling pathway is activated [32, 33]. However, Akt and protein kinase A (PKA) can phosphorylate the Ser-675 site of β-catenin, which contributes to the stability of β-catenin and the regulation to its target gene in the nucleus [33, 34]. Our study shows that insulin treatment activated Akt signaling pathway involving both GSK-3β and β-catenin that leads to activation of the Wnt signaling pathway, which however was attenuated by activation of Nodal-ALK7 signaling. These results are consistent with previous observations that glucagon like peptide-1 and its mimetics promote β-cell proliferation through β-catenin phosphorylation at Ser-675 and activating the Wnt signaling pathway in Akt and PKA dependent fashion [34].

The β-cell compensatory machinery is integrated of positive and negative feedback loops which represents a biosensor to accurately and ultimately sense glucose levels in the blood and consequently release appropriate amounts of insulin to meet body needs [35]. A negative feedback modulator is generally found to be important for maintaining islet mass and/or hormones at appropriate levels [36], and an inadequate feedback suppression is found in obese subjects and is partly account for their prevailing hyperinsulinemia [37]. It is presumably, the antagonistic effects of Nodal and insulin epitomizes such compensatory machinery in the β-cells, however, disruption of the two signals inter-balance may essentially contribute to β-cell dysfunction.

It is noted that the discrepancy of Nodal function in the islets between rodents and humans, in rodent islets Nodal is specifically expressed in the β-cells, whereas in human islets, Nodal is detected in both β-cells and α-cells [38]. Recent study by Boerner et al. demonstrated that Nodal-induced apoptosis only occurred in the α-cell but not in the β-cells of isolated human islets [38], which is not correlated with our finding. Anatomically, human islets contain higher population of α-cells than those in mouse islets [39], and human β-cells are relatively not clustered and mostly associated with α-cells, allowing unique paracrine interactions in human islets [39, 40]. Given that, in rodent islets insulin exerts pro-proliferative effects on the α-cells [41], it is seemingly that the antagonistic effects of Nodal to the paracrine insulin action on the α-cells may result in an attenuated β-cell trophic effects exerted from the α-cells [42–44].

In an in vitro setting, glucose-stimulated insulin secretion was significantly attenuated in INS-1 cells co-treated with Nodal, which was found to be related to its inhibition on Ca^2+^ influx. Our previous studies [9, 10] demonstrated that treatment with Nodal (1 μg/ml) for less than 1 hour would not result in significant INS-1 cell apoptosis. Therefore, Nodal-induced apoptosis alone cannot account for the reduction of insulin secretion observed in this study. Previous study in isolated human islets showed that a chronic exposure of high glucose caused a significant decrease of glucose-stimulated insulin secretion [45]. One possible reason of high glucose attenuated glucose-stimulated insulin secretion is in part through elevation of Nodal signaling in the islet β-cells. In current study we determined that Nodal activation could enhance ALK7–Smad3 signaling pathway, which is in agreement with a negative role for ALK7 in Ca^2+^ signaling and insulin secretion in response to glucose stimulation [15]. Previous studies also demonstrated that disruption of Smad2 in mouse islet β-cells altered ATP-sensitive K^+^ channel activity and insulin secretion [46], and that inhibition of voltage-gated potassium channel signaling up-regulated Smad3 phosphorylation and induction of nuclear activities [47], suggesting that signaling involving Smad proteins affect the secretory machinery in the β-cells. Therefore, the Nodal induced downstream ALK7-Smad3 activation exhibits a negative role in the regulation of β-cell insulin secretion.

## Conclusions

In summary, our study suggest that the antagonistic interaction between Nodal and insulin plays an important role in modulating β-cell mass and secretion. To elucidate the exact signaling mechanism underlying the autocrine Nodal-insulin actions may provide valuable information in developing therapeutic approaches to expand β-cell mass for the treatment of diabetes as a consequence of excessive loss of islet β-cells.

## Additional file

Additional file 1: **Figure S1** Immunocytochemistry of Akt and Smad3 in INS-1 cells treated with culture medium alone or with 1 μg/ml Nodal in the presence or absence of 100 nM insulin for 15 min. Cells were fixed and stained with anti-Akt, anti-p-Akt or anti-Smad3, anti-p-Smad3, and examined by fluorescence microscopy. (PDF 206 kb)

## Abbreviations

BrdU: 5-bromo-2′-deoxyuridine
Co-IP: Co-immunoprecipitation
ERK: Extracellular signal regulated kinase
FBS: Fetal bovine serum
GAPDH: Glyceraldehyde-3-phosphate dehydrogenase
HG: High glucose
ICC: Immunocytochemistry
KRB: Krebs–Ringer bicarbonate
p-Akt: Phosphorylated Akt
Pal: Palmitate
p-ERK1/2: Phosphorylated ERK1/2
p-GSK-3β: Phosphorylated GSK-3β
PI: Propidium iodide
PKA: Protein kinase A
p-Smad3: Phosphorylated Smad3
p-β-cat: Phosphorylated β-catenin
t-Akt: Total Akt
t-caspase-3: Total caspase-3
t-ERK1/2: Total ERK1/2
t-GSK-3β: Total GSK-3β
t-Smad3: Total Smad3
t-β-cat: Total β-catenin
