# Supplementary figures and images for: Antagonistic interaction between Nodal and insulin modulates pancreatic β-cell proliferation and survival
Source: Cell Commun Signal. 2018 Nov 8;16:79. doi: 10.1186/s12964-018-0288-0 (PMC6225724; doi:10.1186/s12964-018-0288-0)

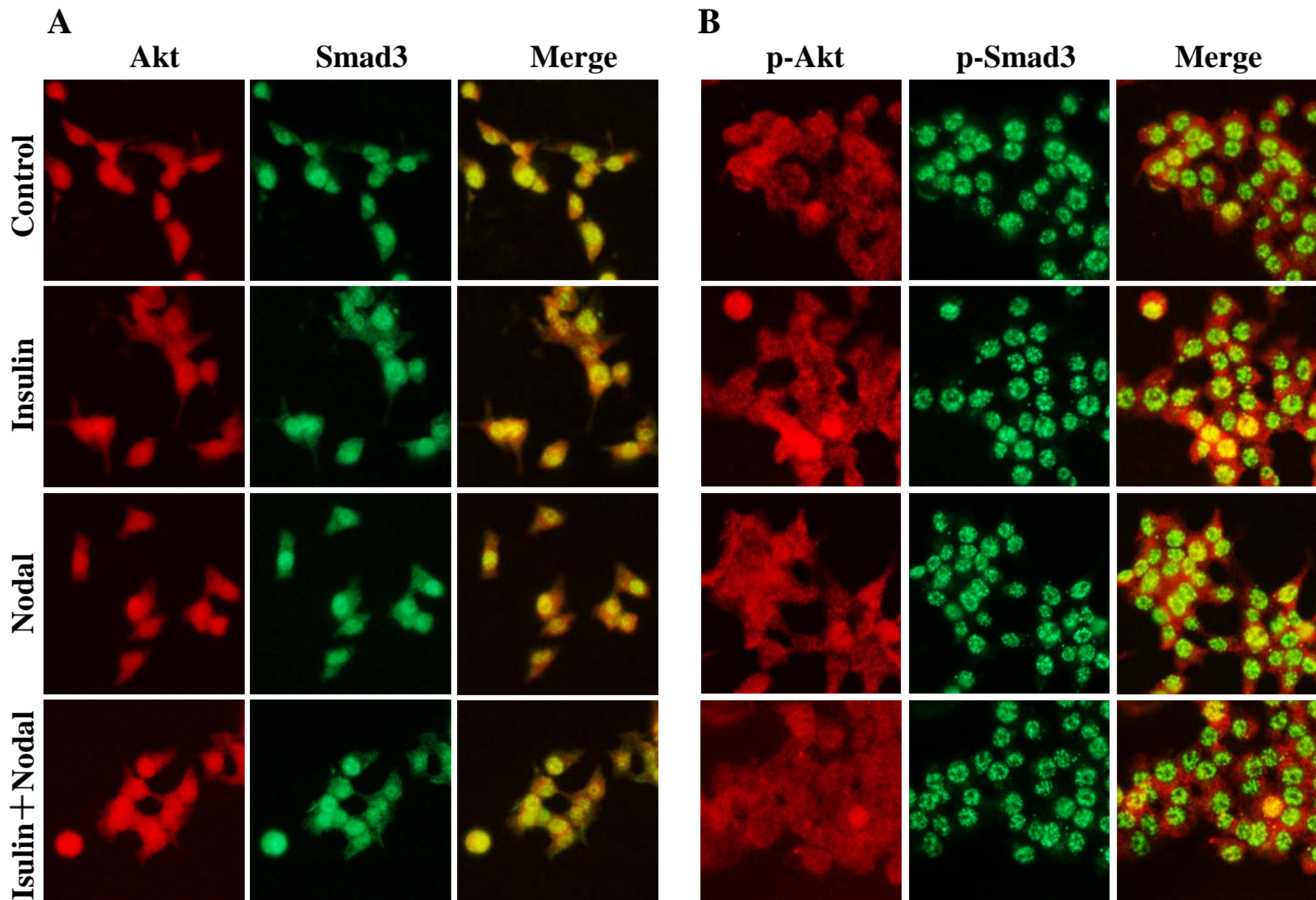

Figure S1

Supplement: Supplementary file 1 — Figure S1 Immunocytochemistry of Akt and Smad3 in INS-1 cells treated with culture medium alone or with 1 μg/ml Nodal in the presence or absence of 100 nM insulin for 15 min. Cells were fixed and stained with anti-Akt, anti-p-Akt or anti-Smad3, anti-p-Smad3, and examined by fluorescence microscopy. (PDF 206 kb) [file 12964_2018_288_MOESM1_ESM.pdf]
